# Primary Macrophage Chemotaxis Induced by Cannabinoid Receptor 2 Agonists Occurs Independently of the CB_2_ Receptor

**DOI:** 10.1038/srep10682

**Published:** 2015-06-02

**Authors:** Lewis Taylor, Ivy Christou, Theodore S. Kapellos, Alice Buchan, Maximillian H. Brodermann, Matteo Gianella-Borradori, Angela Russell, Asif J. Iqbal, David R. Greaves

**Affiliations:** 1 Sir William Dunn School of Pathology, University of Oxford, South Parks Road, Oxford OX1 3RE, UK; 2 Department of Chemistry, Chemistry Research Laboratory, University of Oxford, Mansfield Road, Oxford, OX1 3TA, UK

## Abstract

Activation of CB_2_ has been demonstrated to induce directed immune cell migration. However, the ability of CB2 to act as a chemoattractant receptor in macrophages remains largely unexplored. Using a real-time chemotaxis assay and a panel of chemically diverse and widely used CB_2_ agonists, we set out to examine whether CB_2_ modulates primary murine macrophage chemotaxis. We report that of 12 agonists tested, only JWH133, HU308, L-759,656 and L-759,633 acted as macrophage chemoattractants. Surprisingly, neither pharmacological inhibition nor genetic ablation of CB_2_ had any effect on CB_2_ agonist-induced macrophage chemotaxis. As chemotaxis was pertussis toxin sensitive in both WT and CB_2_^-/-^ macrophages, we concluded that a non-CB_1_/CB_2_, G_i/o_-coupled GPCR must be responsible for CB_2_ agonist-induced macrophage migration. The obvious candidate receptors GPR18 and GPR55 could not mediate JWH133 or HU308-induced cytoskeletal rearrangement or JWH133-induced β-arrestin recruitment in cells transfected with either receptor, demonstrating that neither are the unidentified GPCR. Taken together our results conclusively demonstrate that CB_2_ is not a chemoattractant receptor for murine macrophages. Furthermore we show for the first time that JWH133, HU308, L-759,656 and L-759,633 have off-target effects of functional consequence in primary cells and we believe that our findings have wide ranging implications for the entire cannabinoid field.

The cannabinoid receptors, CB_1_ and CB_2_, received their name from the discovery that they are activated by Δ9-tetrahydrocannabinol (THC), the major psychoactive component of cannabis^1^. These G protein-coupled receptors (and potentially other putative GPCRs^2^), in combination with their endogenous ligands (the endocannabinoids) and the enzymes that synthesise and degrade these cognate lipids, comprise the endocannabinoid system^3^. Both CB_1_ and CB_2_ are G_i/o_-coupled, therefore their activation results in adenylyl cyclase inhibition and decreased intracellular cAMP^4^. Moreover, both receptors can initiate additional downstream signalling events, including activation of intracellular kinases and voltage gated Ca^2+^ channels^5^,^6^. Whilst CB_1_ is predominantly expressed throughout the brain^7^, CB_2_ is primarily localised on cells of the immune system^8^.

This expression profile led to the hypothesis that CB_2_ acts as an immunomodulatory receptor. Indeed, CB_2_ has been shown to modulate multiple inflammatory diseases and immune cell functions^9^,^10^,^11^, including directed migration or chemotaxis^12^. Activation of CB_2_ has been demonstrated to elicit leukocyte chemotaxis as the synthetic and highly potent CB_2_ agonists JWH015 and JWH133 cause human monocyte migration^13^ and the endocannabinoid 2-arachidonylglycerol (2-AG) induces the directed migration of B lymphocytes^14^, natural killer cells^15^, eosinophils^16^, the myeloid HL-60 cell line and human monocytes^17^. In all cases SR144528 (a CB_2_ inverse agonist) inhibited 2-AG-induced chemotaxis, demonstrating dependence on CB_2_ signalling.

However, it is becoming increasingly apparent that CB_2_ plays a complex role in the modulation of leukocyte chemotaxis. Although 2-AG acts as a chemoattractant, the mixed CB_1_/CB_2_ agonists WIN,55212-2 and CP55,940 fail to elicit directed cellular migration^14^,^17^, hinting that functional selectivity may impact CB_2_-mediated chemotaxis. This phenomenon, also known as biased agonism, is defined as the ability of different ligands at the same receptor to activate distinct downstream signalling pathways^18^ and has already been documented for ligands acting at CB_2_^19^,^20^. However as previous chemotaxis studies only use a limited range of CB_2_ agonists, the extent and importance of functional selectivity within CB_2_-mediated chemotaxis is unknown. Furthermore, whether CB_2_ acts as a chemoattractant receptor in macrophages remains largely unexplored. As these innate immune cells play a core role within the induction and continuation of an inflammatory response, they are central to many chronic inflammatory diseases^21^,^22^. Therefore understanding the mechanisms that regulate macrophage dynamics and migration are of great interest.

Previous work has demonstrated that the activation of CB_2_ may negatively regulate macrophage migration to other chemotactic factors. CP55,940, alongside the phytocannabinoid cannabidiol, inhibits the migration of rat macrophages towards the chemoattractant peptide N-formyl-methionyl-leucyl-phenylalanine (fMLP)^23^,^24^. Furthermore, Steffens *et al.* demonstrated that Δ9-THC treatment of murine macrophages inhibits their chemotaxis towards CCL2^25^, whilst a parallel report by Raborn *et al.* found that Δ9-THC and CP55,940 also inhibit macrophage migration towards CCL5^26^. However, this negative role seems to be at odds with CB_2_ behaving as a chemoattractant receptor in other leukocyte populations and further complexity is added by the finding that, in contrast to 2-AG, JWH015, CP55,940 and Δ9-THC are unable to inhibit neutrophil chemotaxis toward fMLP^27^,^28^, suggesting that functional selectivity may also impact this process as well.

With this in mind, we aimed to use a panel of commercially available and chemically diverse CB_2_ agonists to elucidate whether CB_2_ is a chemoattractant receptor in primary murine macrophages and assess the contribution of functional selectivity. We found that of twelve CB_2_ agonists tested, only JWH133, HU308, L-759,656 and L-759,633 induced macrophage cytoskeletal rearrangement and chemotaxis. Although chemotaxis was pertussis toxin sensitive, genetic ablation of CB_2_ had no effect on CB_2_ agonist-induced macrophage signalling or chemotaxis. Therefore, we conclude that CB_2_ is not a chemoattractant receptor in murine macrophages, and that chemotaxis elicited by CB_2_ agonists occurs via an off-target effect at a non-CB_1_/CB_2_ G_i/o_-coupled GPCR.

## Materials and Methods

### Reagents

Cannabinoids and cannabinoid receptor inverse agonists were purchased from Tocris (Bristol, UK). Murine CCL5 was purchased from Peprotech (London, UK). Murine chemerin and murine CCL2 were obtained from R & D systems (Abingdon, UK). Pertussis toxin (PTX) was purchased from Merck Millipore (Feltham, UK). Bio-gel polyacrylamide beads (P-100 fine 45-90 μm) were purchased from Biorad (Hertfordshire, UK). cOmplete, EDTA free protease inhibitor cocktail tablets were purchased from Roche (Burgess Hill, UK). Rabbit anti-phospho-ERK1/2 and total ERK1/2 were purchased from Cell Signalling Technologies (Danvers, MA, USA). HRP-conjugated Goat anti-rabbit secondary was purchased from Biorad. CIM-16 plates were purchased from Cambridge bioscience (Cambridge, UK). All cell culture media were obtained from PAA systems (Yeovil, UK). Phosphatase inhibitor cocktail 2 and all other laboratory chemicals were purchased from Sigma Aldrich (Dorset, UK). DIAS2 (3-((2’-cyanobenzyl)thio)-5*H*-[1,2,4]triazino[5,6b]indole) was synthesized by Dr. Angela Russell (University of Oxford, UK)

### Animals

C57BL6/J mice were obtained from Harlan Laboratories (Oxfordshire, UK). Cannabinoid receptor 2 knockout mice (herein referred to as CB_2_^-/-^ mice) were purchased from the Jackson Laboratories (Bar harbour, ME, USA). These mice were originally produced by Deltagen (San Mateo, CA, USA) and have been backcrossed at least 5 generations onto the C57BL6/J background. All animal studies were conducted with ethical approval from the Dunn School of Pathology Local Ethical Review Committee and in accordance with the UK Home Office regulations (Guidance on the Operation of Animals, Scientific Procedures Act, 1986)

### Generation of N-terminal tagged GPCR constructs

All tagged GPCR constructs were purchased from Life Technologies, using their GeneArt® Gene Synthesis service. Briefly, the protein coding regions for murine C5aR1 (Transcript variant 1, NM_007577.4), CB_2_ (NM_009924.3), GPR55 (NM_001033290.2), and GPR18 (NM_182806.1) were modified by the removal of the endogenous start codon and the addition of an N-terminal EcoRI restriction site, a Kozak consensus sequence, an epitope tag (C5aR1 and GPR18 – hemagglutinin (HA); CB_2_ – FLAG; GPR55 – c-myc) and a C-terminal NotI restriction site. The sequences were cloned into pcDNA3.1(+) and all constructs 100% sequence verified. Vectors were propagated by transformation into DH5α chemically competent cells (Life Technologies) following the manufacturers protocol. After being spread on LB agar plates containing 50 μg/ml ampicillin and incubated overnight at 37 °C, single colonies were placed into 7 ml of LB medium containing 50 μg/ml ampicillin and incubated for 10 hours at 37 °C with constant shaking (300 rpm). Next 150 μl of this culture was added to 150 ml LB medium containing 50 μg/ml ampicillin and incubated for 20 hours at 37 °C with constant shaking (300 rpm). The cells were then pelleted and plasmid DNA extracted using the Qiagen HiSpeed plasmid midi kit following the manufacturer’s instructions. DNA concentration and purity was determined using a NanoDrop™ ND-1000 spectrophotometer (Thermo Scientific).

### Bio-gel and thioglycollate elicitation of peritoneal exudate cells (PECs)

Male C57BL/6J mice or male and female CB_2_^-/-^ mice were injected intraperitoneally with either 1 ml of sterile 2% bio-gel polyacrylamide beads (P100 fine, 45-90 μM) suspended in phosphate buffered saline (PBS) or 1 ml of 4% thioglycollate (brewer thioglycollate media, Sigma-Aldrich). After four days, mice were sacrificed and the elicited cells collected by peritoneal lavage with 10 ml ice cold PBS containing 2 mM EDTA.

### PEC macrophage enrichment

Following peritoneal lavage bio-gel elicited PECs were pelleted by centrifugation at 200 × g for 5 min at 4 °C and then re-suspended in 10 ml chemotaxis buffer (RPMI 1640 supplemented with 0.5% BSA and 25 mM HEPES) before being transferred into 10 cm petri dishes (one per mouse; non-tissue culture treated) and left for 2 hours at 37 °C, 5% CO_2_. After three washes with 10 ml PBS to remove any non-adherent cells, the medium was replaced with 10 ml chemotaxis buffer and dishes left overnight at 37 °C, 5% CO_2_. Adherent macrophages were collected by the addition of PBS, containing 10 mM EDTA, and gentle agitation. Macrophages were then pelleted by centrifugation at 200 × g for 5 min at 4 °C, resuspended in chemotaxis buffer, counted and then adjusted to the desired cell concentration. Macrophage purity was determined by flow cytometry as described below.

### Modified Boyden chamber chemotaxis

Following peritoneal lavage, bio-gel elicited PECs were pelleted by centrifugation at 200 × g for 5 min at 4 °C and then re-suspended at a cell density of 5 × 10^6^ cells/ml in chemotaxis buffer pre-warmed to 37 °C. Cannabinoid or vehicle (325 μl - 0.3% DMSO in chemotaxis buffer) was added to the lower chamber of a 96-well Neuroprobe ChemoTx plate (8 μm pore size – Receptor technologies, Leamington Spa, UK). Following attachment to the plate, 80 μl of cell suspension (4 × 10^5^ cells) was added onto the filter site above each well. The plate was then incubated for 4 hours at 37 °C, 5% CO_2_. The assay was terminated by detaching the filter and wiping to remove non-migrated cells from the filter top. Migrated cells were then fixed with 1% formalin in PBS for 10 min, rinsed with PBS and then DAPI-stained (1 μg/ml in distilled water) for 10 min in the dark. Following a final rinse with PBS, the filter sites were cut, mounted using fluorescent mounting medium, and then left overnight at 4 °C. The following day, DAPI-stained nuclei were detected using an Axiovert 200 inverted fluorescence microscope (Zeiss, Cambridge, UK) and two images taken per filter site. Metamorph software (Molecular Devices, Wokingham, UK) was used to count the number of nuclei in each image. Migration index (MI) was calculated by dividing the number of migrated cells for each treatment by the mean of the number of cells migrated towards vehicle alone.

### ACEA xCELLigence chemotaxis assay

Real-time chemotaxis analysis was conducted as previously described^29^. Briefly, vehicle (0.3% DMSO in chemotaxis buffer), cannabinoid, chemerin or chemokine (160 μl) at the desired final concentration was added to the lower chamber of a CIM-16 plate. The upper chamber was subsequently attached and 50 μl of pre-warmed chemotaxis buffer added to each of the upper chambers. Following equilibration at RT for 30 min, the plate was transferred into the RTCA-DP system for background analysis. Bio-gel elicited PECs (50 μl - 4 × 10^5^ cells/well), enriched macrophages, thioglycollate elicited macrophages or bone marrow neutrophils (50 μl - 2 × 10^5^ cells/well) in chemotaxis buffer were then added to all upper wells and plate replaced into the RTCA-DP system. Cell Index (CI) measurements were then taken every 5 seconds over the 3-4 hour assay period. Migration Index (MI) was calculated as maximum cell index minus minimum cell index and pooled data are displayed as a fold change relative to cells migrating towards vehicle alone.

### ACEA 96 well ECIS assay

Following peritoneal lavage, bio-gel elicited PECs were pelleted by centrifugation at 200 × g for 5 min at 4 °C and then re-suspended at a cell density of 1 × 10^6^ cells/ml in chemotaxis buffer pre-warmed to 37 °C. Chemotaxis buffer (50 μl) was then added into all wells of a 96 well E-plate and a background measurement taken. Afterwards, 50 μl of cell suspension was added to all wells (50,000 cells/well) and cells were then left at 37 °C, 5% CO_2_ for 2-3 hours to adhere until the CI had reached a stable plateau. Cells were then stimulated with either vehicle (0.3% DMSO) or cannabinoid at the indicated concentration and CI measurements taken every 5 seconds for 1-2 hours after agonist addition. Data are displayed as change in CI from the point of agonist addition (Δ Cell Index) and response was calculated as maximum CI – CI at point of agonist addition (Δ Cell Index (max-min)). For the receptor transfection studies, transfected CHO cells were plated into a 96 well E-plate (50 μl; 25,000 cells/well) in Ham’s F12 with 2% FCS and left overnight at 37 °C, 5% CO_2_ to reach a stable baseline and allow tagged GPCR expression. After stimulation with ligand (Cannabinoid 10 μM, C5a 10 nM or vehicle (0.3% DMSO)), CI measurements were taken every 5 seconds for 1-2 hours after agonist addition. Response was calculated as: CI immediately before agonist addition – CI at the time point corresponding to C5a peak in C5aR1 transfected cells.

### Intracellular cAMP measurement

Intracellular cAMP levels were measured using Discoverx cAMP Hunter™ eXpress kits (Discoverx, Birmingham, UK) following the manufacturer’s protocol. Briefly, CHO-K1 cells overexpressing the human CB_2_ receptor were plated into a ½ area 96 well plate (15,000 cells/well) and incubated at 37 °C, 5% CO_2_ for 24 hours. The media was then removed and replaced with 22.5 μl of assay buffer containing a cAMP capture antibody. Cells were then stimulated for 30 min at 37 °C, 5% CO_2_ with either vehicle (4.8% DMSO with 80 μM forskolin) or cannabinoid at 4x the final desired concentration (Final concentrations were 1.2% DMSO and 20 μM forskolin). Following stimulation, cell lysis and cAMP detection were performed as per the manufacturer’s protocol. Luminescence measurements were taken using a PHERAstar microplate reader (BMG Labtech, Aylesbury, UK).

### Cannabinoid stimulation of bio-gel elicited murine macrophages

Following peritoneal lavage, bio-gel elicited PECs were pelleted by centrifugation at 200 xg for 5 min at 4 °C and then re-suspended at a cell density of 1 × 10^6^ cells/ml in chemotaxis buffer pre-warmed to 37 °C. PECs (2 × 10^6^ cells/well) were then seeded in 6 well plates and allowed to adhere for 2 hours at 37 °C, 5% CO_2_. After three washes with pre-warmed PBS to remove non-adherent cells and enrich for macrophages, 2 ml of chemotaxis buffer was added to each well and the plates left for 1 hour at 37 °C, 5% CO_2_. The media was then replaced with 1 ml warmed chemotaxis buffer and left for a further hour at 37 °C, 5% CO_2_, after which 200 μl of vehicle (1.8% DMSO in chemotaxis buffer) or 60 μM cannabinoid was added to the corresponding wells (yielding a final concentration of 10 μM at 0.3% DMSO). Plates were then incubated for 30 min at 37 °C, 5% CO_2_. For SR144528 studies, 100 μl of either vehicle (3.3% DMSO) or 11 μM SR144528 was added to the corresponding wells and plates incubated for 30 min at 37 °C, 5% CO_2_ (yielding a final concentration of 1 μM SR144528 at 0.3% DMSO). Following antagonist pre-treatment, wells received 100 μl of either vehicle (3.9% DMSO in chemotaxis buffer) or 120 μM cannabinoid (yielding final concentrations of 10 μM cannabinoid and 0.6% DMSO) and plates incubated for 30 min at 37 °C, 5% CO_2_. After stimulation, the media was rapidly removed and the plates placed at −80 °C. Cell lysates were prepared by the addition to each well of 180 μl cell lysis buffer (150 mM NaCl, 0.8 mM MgCl_2_, 5 mM EGTA, 50 mM HEPES, 1% IGEPAL CA-630) supplemented with protease and phosphatase inhibitors. Cells were then manually disrupted and supernatant centrifuged at 16,000 × g for 10 min at 4 °C. Protein concentration of cell debris free supernatant was determined using a BCA protein assay kit (Thermo Fisher scientific, Loughborough, UK) following the manufacturer’s protocol. Samples were then diluted 3:1 with 4x laemmli buffer (250 mM Tris-HCl, pH 6.8, 8% SDS, 40% glycerol, 0.004% bromophenol blue, 20% β-mercaptoethanol), heated at 95 °C for 5 min and either loaded directly onto an SDS-PAGE gel or placed at −80 °C.

### Phosphorylated and total ERK1/2 western blotting

Samples (30 μg total protein) were separated using a 10% SDS-PAGE gel and transferred onto Hybond ECL nitrocellulose (GE healthcare, Buckinghamshire, UK). Membranes were blocked with 5% BSA in TBS-T for 2 hours at RT or overnight at 4 °C. After blocking, membranes were incubated with rabbit anti-phospho-ERK1/2 (1:2000) diluted in 5% BSA/TBS-T for 2 hours at RT or overnight at 4 °C. Membranes were then incubated with a HRP-conjugated anti-rabbit secondary antibody (1:20,000) for 1 hour at RT. Protein bands were visualised by incubating the membranes for 5 min with Amersham™ ECL prime and subsequent exposure to x-ray film over a range of exposure times. To confirm equal protein loading between samples, bound antibodies were removed by incubating the nitrocellulose membranes in stripping buffer (60 mM Tris-HCl pH 6.8, 2% SDS, 0.8% β-mercaptoethanol) for 30 min at 50 °C. Membranes were then blocked with 5% BSA in TBS-T for 2 hours at RT and then incubated with rabbit anti-ERK1/2 (1:2000) diluted in 5% BSA/TBS-T for 2 hours. Protein band detection was conducted as described above. Densitometric analysis was conducted using Image Studio Lite (LI-COR Biosciences, Cambridge, UK) with data displayed as fold change compared to vehicle of phospho-ERK1/2 band density divided by total ERK1/2 band density.

### Flow cytometry

Cells (5 × 10^5^) were placed into polypropylene tubes and pelleted by centrifugation at 200 × g for 5 mins at 4 °C. These were then resuspended in FACS buffer (PBS containing 2% FCS, 25 mM HEPES and 5 mM EDTA) supplemented with mouse IgG and mouse SeroBlock FcR^®^ (AbD Serotec, Oxford, UK) and left on ice for 30 min. For studies using CHO cells, the cell pellet was resuspended in FACS buffer alone. Specific staining was conducted using the following antibodies and appropriate isotype controls: F4/80 (1:100, FITC, clone CI:A3-1, Abcam, Cambridge, UK), Ly-6B.2 (1:100, Alexa Fluor® 647, clone 7/4, Abd Serotec), Ly-6G (1:100, PE, clone 1A8, BD Biosciences, Oxford, UK), CD11b (1:100, FITC, clone M1/70, BD Biosciences) anti-HA (1:50, APC, clone GG8-1F3.3.1, Miltenyi Biotec, Surrey, UK), anti-c-myc (1:10, FITC, clone SH1-26E7.1.3, Miltenyi Biotec), anti-FLAG (anti-DYKDDDDK, 1:50, PE, clone L5, Biolegend, London, UK) for 1 hour on ice. Cells were then pelleted by centrifugation at 200 × g for 5 mins at 4 °C, resuspended in 1% formaldehyde and analysed using a Dako Cyan ADP flow cytometer (Beckman Coulter Ltd, High Wycombe, UK) and FlowJo software (V10, Tree Star, Ashland, USA).

### RNA extraction and reverse transcription

RNA extraction of enriched bio-gel macrophages was conducted using the RNeasy® Mini Kit (Qiagen, Manchester, UK) following the manufacturer’s instructions. RNA concentration and quality was determined using a NanoDrop™ ND-1000 spectrophotometer. cDNA was produced from the purified RNA using the QuantiTect® Reverse Transcription kit (Qiagen) following the manufacturer’s protocol.

### Real-time PCR

Expression of actin, CB_2_, GPR55 and GPR18 in enriched bio-gel macrophages was determined using the following primers (5’>3’): Actin Fwd CCAACAGCAGACTTCCAGGATT, Actin Rev CTGGCAAGAAGGAGTGGTAACTG, CB_2_ Fwd GGTCCTCTCAGCATTGATTTC, CB_2_ Rev GCCCAGTAGGTAGTCGTTAG, GPR55 Fwd AACCTTCATCGGCTCCTCT, GPR55 Rev ATTCTTCCTGTCCCACTCCT, GPR18 Fwd CGACCAAGAAAAGAACCACAG, GPR18 Rev AATGAAAGCAAGAAGCCACA and SYBR Select PCR master mix (Life Technologies). 500 ng of cDNA was used per reaction with the following thermal profile: 95 °C for 5 min, 40 cycles of 95 °C for 30 s, 60 °C for 20 s, 72 °C for 30 s and a final step of 72 °C for 5 min using a RG-3000 thermal cycler (Corbett research, Manchester UK). Analysis was conducted using Rotor-gene 6 software.

### CHO cell GPCR transfections

CHO cells were cultured in Ham’s F12 medium containing 2% FCS until 80-90% confluent. After washing twice with 15 ml PBS, cells were detached by incubation with TrypLE™ Express (5 ml - T75 flask, 10 ml - T175 flask, Life technologies, Paisley, UK) for 3 min at 37 °C, 5% CO_2_. After being collected into 25 ml Ham’s F12 with 2% FCS and pelleted by centrifugation at 200 × g at 4 °C, cells were resuspended in Ham’s F12 with 2% FCS, counted and cell density adjusted to 1 × 10^6^ cells/ml. For receptor transfections, the Amaxa^®^ cell line nucleofector^®^ kit T was used following the manufacturers recommended protocol for CHO cells. Briefly, 1 × 10^6^ cells were pelleted by centrifugation at 200 × g for 10 min at RT and then resuspended in 100 μl nucleofector^®^ solution T. After addition of Plasmid DNA (2 μg - either tagged GPCR or empty vector) the suspension was transferred into a provided cuvette, placed into the nucleofector^®^ device and transfected using program U-023. Cells were then made to a final volume of 2 ml using Ham’s F12 with 2% FCS and plated into a 96 well E-plate (see above protocol for details). The remaining cells were added into a 6 well dish containing 2 ml Ham’s F12 with 2% FCS and left overnight at 37 °C, 5% CO_2_ for flow cytometric analysis of transfection efficiency. Cells were detached by incubation with 500 μl TrypLE™ Express per well for 3 min at 37 °C, 5% CO_2_. After collection into 5 ml Ham’s F12 with 2% FCS, cells were pelleted, counted and then used for flow cytometry as detailed above.

### Discoverx GPCR β-arrestin recruitment screening

Recruitment of β-Arrestin to 241 human GPCRs after stimulation with 10 μM JWH133 was measured using the Discoverx PathHunter® eXpress β-Arrestin GPCR Assay following the manufacturer’s protocol. Briefly, cells were seeded into white walled, 384 well microplates and incubated at 37 °C for the appropriate time period prior to testing. JWH133 or vehicle (1% DMSO) was then added to the corresponding wells and the plate incubated at either RT or 37 °C for 90 or 180 min, depending on the GPCRs being tested. Cell lysis and detection reagents were subsequently added and one hour later, luminescence measurement taken using a PerkinElmer Envision microplate reader. For the GPCRMax panel, % Agonist activity is calculated as 100% x (mean of test sample - mean of vehicle control) / (mean MAX control ligand - Mean of vehicle control). For the OrphanMax panel, % Agonist activity is calculated as 100% x (mean of test sample - mean of vehicle control) / (mean of vehicle control). A complete list of GPCRs tested can be found as Supplementary data S1 online.

## Results

### The CB_2_ selective agonists JWH133 and HU308 are chemoattractants for bio-gel elicited PECs

**Figure 1:**
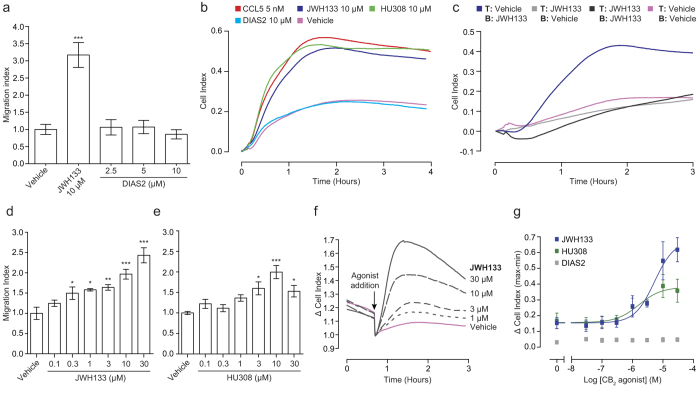
The CB_2_ selective agonists JWH133, HU308 and DIAS2 differ in their ability to induce the directional migration of murine PECs. (**A**) Bio-gel elicited murine PECs were placed into the top well of a modified 96 well Boyden chamber (8 μm pore size) and allowed to migrate towards vehicle or cannabinoid for 4 hours at 37 °C, 5% CO_2_. JWH133 (10 μM) significantly induced macrophage chemotaxis; however DIAS2 had no effect on macrophage migration. Data are mean± SEM, n = 3 biological replicates. (**B**-**E**) Bio-gel elicited murine PECs were placed into the upper chamber of a CIM-16 plate and allowed to migrate for 3-4 hours at 37 °C, 5% CO_2_ towards indicated compounds. (**B**) Real-time chemotaxis analysis confirmed that JWH133 and HU308, but not DIAS2 (all 10 μM), elicit PEC chemotaxis. (**C**) Chemokinetic analysis, comparing all combinations of vehicle or cannabinoid (10 μM) in the top (T) and bottom (B) chambers, demonstrated that JWH133 induced true chemotaxis. Data are mean real-time traces from one experiment with 4 technical replicates per condition, and are representative of 2 independent biological replicates. (**D**,**E**) Concentration response analysis found that both JWH133 and HU308 elicit PEC chemotaxis in a concentration dependent manner. Data are mean ± SEM, n = 4 biological replicates with 3-4 technical replicates per condition. (**F**,**G**) Bio-gel elicited PECs were placed into a 96 well E-plate allowed to adhere for 2-3 hours at 37 °C, 5% CO_2_. Afterwards, cells were stimulated with either vehicle or cannabinoid at the indicated concentration. (**F**) Representative raw traces showing cell responses to JWH133 (30, 10, 3 and 1 μM). Data are mean traces from one experiment with 2-3 technical replicates per condition. (**G**) Concentration response quantification demonstrates that JWH133 and HU308 caused macrophage cytoskeletal rearrangement in a concentration dependent manner, with EC_50_ values of 5.3 and 1.6 μM. Data are mean ± SEM, n = 3 biological replicates with 2-3 technical replicates per condition. Statistical analysis was conducted by one-way ANOVA with Dunnett’s multiple comparisons correction. * P < 0.05, ** P < 0.01 *** P < 0.001, comparing all samples to vehicle control.

To initially address whether CB_2_ selective agonists are capable of inducing directed migration of murine PECs, we utilised the conventional modified Boyden chamber chemotaxis assay. Figure 1A shows that the classical, widely used and potent CB_2_ agonist JWH133 significantly induced PEC migration (P < 0.001 compared to vehicle alone). In contrast, our own novel and selective CB_2_ agonist, DIAS2^30^, did not induce PEC chemotaxis regardless of the concentration used. DIAS2 had no effect on cell viability at all of the concentrations used (data not shown), ruling out toxicity as an explanation for the absence of migration.

As a chemotaxis methodology the modified Boyden chamber has a number of limitations, chief among these being the ability to only measure migration at a single time point. To overcome this issue, we employed a novel chemotaxis technology that allows cellular migration to be followed in real-time. This is achieved by measuring electrical impedance across a gold electrode that covers the underside of a Boyden chamber-style filter. This impedance is converted into a parameter known as Cell Index (CI), with increasing CI equating to higher numbers of migrated cells^29^. Real-time chemotaxis confirmed our initial Boyden chamber results, with JWH133 (10 μM) eliciting a ~ 2 fold increase in CI versus vehicle alone, with peak CI reached at ~ 1.5 hours. DIAS2 (10 μM) remained negative, displaying similar kinetics to vehicle alone (Fig. 1B). Alongside JWH133, we also examined whether HU308, a non-classical CB_2_ agonist, could also act as a chemoattractant for murine PECs. Real-time analysis showed that HU308 (10 μM) did indeed cause chemotaxis with similar kinetics and efficacy to JWH133 (Fig. 1B). Compared to the chemokine CCL5 (5 nM), both JWH133 and HU308 displayed similar chemotaxis kinetics and maximal responses, albeit with reduced potency (Fig. 1B). Further analysis of these ligands demonstrated that JWH133 did not induce fugetaxis (migration away from a soluble ligand - top chamber: 10 μM cannabinoid, bottom chamber: vehicle) or chemokinesis (non-directional migration stimulated by a soluble ligand - 10 μM cannabinoid in top and bottom chamber), as migration kinetics under these conditions closely mimicked vehicle alone (Fig. 1C). Analogous results were also obtained with HU308 (data not shown). Moreover, JWH133 and HU308 induced chemotaxis in a concentration dependent manner (Fig. 1D,E, respectively). Taken together, these experiments demonstrate that JWH133 and HU308 are true chemoattractants for primary murine PECs, whereas our novel CB_2_ agonist, DIAS2, is not.

In order to corroborate these findings, we employed another impedance based assay that monitors cytoskeletal rearrangements which occur downstream of receptor activation in real-time^31^,^32^,^33^, thereby providing an independent, but complementary, functional readout to chemotaxis. The representative traces shown in Fig. 1F demonstrate that JWH133 elicited concentration dependent increases in CI. Concentration response analysis determined EC_50_ values of 5.3 and 1.6 μM for JWH133 and HU308, respectively (Fig. 1G). DIAS2 was negative at all concentrations tested, corroborating the chemotaxis results (Fig. 1G). In summary, only the chemotaxis positive compounds were capable of inducing changes in cell morphology as measured by changes in electrical impedance.

### Macrophages are the predominant population in bio-gel elicited PECs that are being measured in real-time chemotaxis

**Figure 2:**
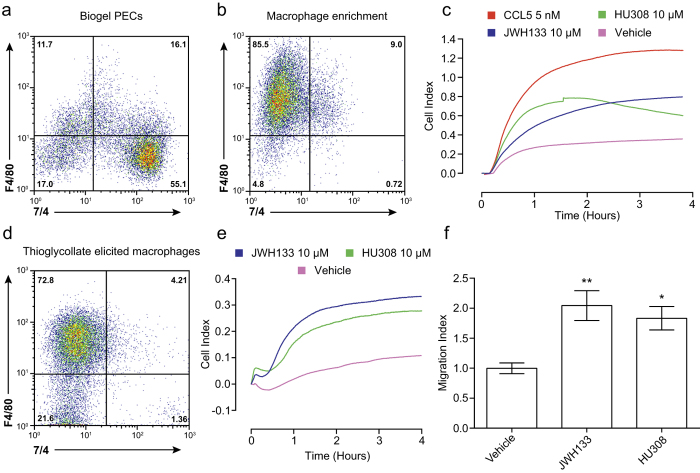
Macrophages are the dominant cell population in bio-gel elicited PECs that migrate toward CB_2_ agonists. (**A**) Flow cytometry analysis demonstrates that neutrophils (F4/80^-^, 7/4^+^) are the major cell population within bio-gel elicited PECs, whereas macrophages (F4/80^+^, 7/4^-^) and monocytes (F4/80^+^, 7/4^+^) form only a minor population. (**B**) Bio-gel elicited PECs were placed into petri dishes and allowed to adhere for 2 hours at 37 °C, 5% CO_2_ prior to washing with PBS. Cells were then left overnight at 37 °C, 5% CO_2_ and remaining adherent cells removed using PBS containing 10 mM EDTA. Flow cytometry analysis of these cells after staining with F4/80 and 7/4 demonstrates that this process strongly enriches macrophages and removes neutrophils. (**C**) Enriched macrophages (2 × 10^5^) were placed into the upper chamber of a CIM-16 plate and allowed to migrate for 4 hours at 37 °C, 5% CO_2_ toward vehicle (0.3% DMSO), 5 nM chemokine or 10 μM cannabinoid. Real-time chemotaxis analysis demonstrates that JWH133 and HU308 caused strong directed migration of this macrophage population. Data show mean trace of 3-4 technical replicates per condition. (**D**) Flow cytometry analysis using F4/80 and 7/4 demonstrates that macrophages are the main cell population elicited by intraperitoneal injection of 4% thioglycollate. (**E**,**F**) Thioglycollate elicited macrophages (2 × 10^5^) were placed into the upper chamber of a CIM-16 plate and allowed to migrate for 4 hours at 37 °C, 5% CO_2_ toward vehicle (0.3% DMSO) or 10 μM cannabinoid. (**E**) Representative real-time chemotaxis trace of thioglycollate elicited macrophage migration towards JWH133, HU308 or vehicle. Data show mean trace of 3-4 technical replicates per condition. (**F**) Quantification of chemotaxis demonstrates that JWH133 and HU308 significantly induced thioglycollate elicited macrophage chemotaxis. Data are mean ± SEM, n = 4-6 biological replicates with 3-4 technical replicates per condition. Statistical analysis was conducted by one-way ANOVA with Dunnett’s multiple comparisons correction. * P < 0.05, ** P < 0.01, comparing all samples to vehicle control.

We initially selected bio-gel as our eliciting agent as the literature suggests that this method allows recovery of large cell numbers with 50-60% macrophage purity^34^. However, after analysing the cell populations elicited by bio-gel using flow cytometry, we reproducibly found that neutrophils (F4/80^-^,7/4^+^) were the predominant cell population (Fig. 2A). Both macrophages (F4/80^+^,7/4^-^) and monocytes (F4/80^+^,7/4^+^) formed minor populations and the large neutrophil population persisted when a different batch of bio-gel or animals housed in a different facility were used (data not shown). However, neutrophils are unlikely to contribute to the observed CI signal when using bio-gel PECs in the real-time chemotaxis system, as migration of highly pure bone marrow neutrophils towards the anaphylatoxin C5a, a well know neutrophil chemoattractant^35^, yields an absolute CI signal of less than 0.08 (data not shown). It is important to note that this does not mean neutrophils do not migrate towards the cannabinoids, it simply means that these cells are not measured by the real-time system and can therefore be discounted as a population that contributes to the CI observed. Instead, real-time chemotaxis analysis of macrophages enriched from bio-gel PECs by overnight plating (~85% macrophage and <1% neutrophil; Fig. 2B), demonstrated that both JWH133 and HU308 (10 μM) robustly induced directed migration of this enriched macrophage population. CCL5 (5 nM) was used as a positive control. To further probe the ability of macrophages to migrate towards JWH133 and HU308, we next used thioglycollate elicitation that yielded a large macrophage population (~70%) with minimal neutrophils (Fig. 2D). Quantification of real-time chemotaxis found that JWH133 and HU308 significantly induced thioglycollate elicited macrophage chemotaxis (Fig. 2F; P = 0.0024 and 0.00161, respectively). A representative real-time chemotaxis trace is shown in Fig. 2E. Together this data demonstrates that macrophages can migrate towards JWH133 and HU308 and these are the predominant population in bio-gel elicited PECs that are being measured in real-time chemotaxis analysis.

### Only a subset of CB_2_ agonists act as chemoattractants for murine macrophages

**Figure 3:**
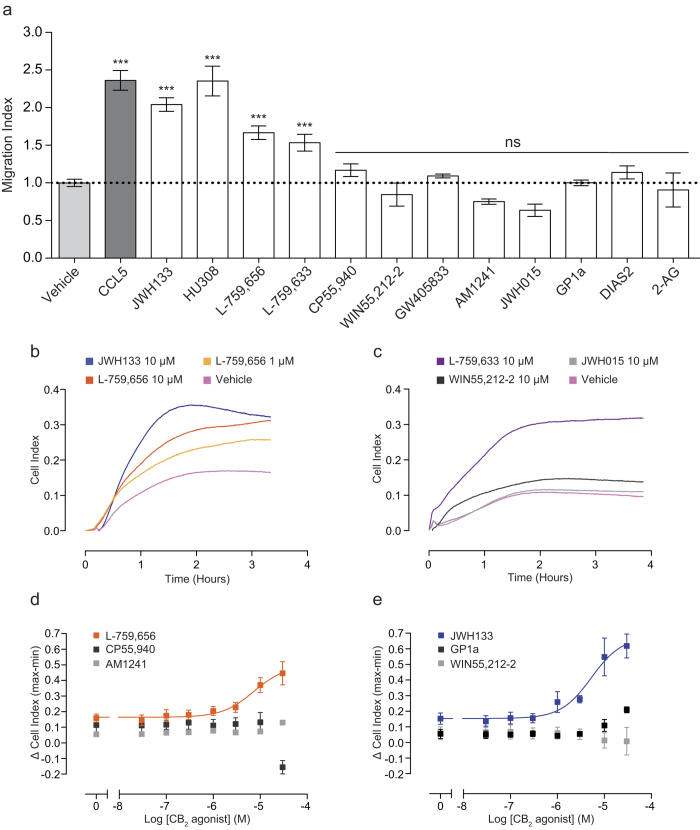
Only a subset of CB_2_ agonists are primary macrophage chemoattractants. (**A**-**C**) Bio-gel elicited murine PECs (4 × 10^5^) were placed into the upper chamber of a CIM-16 plate and allowed to migrate for 3-4 hours at 37 °C, 5% CO_2_ toward vehicle (0.3% DMSO), 5 nM chemokine or 10 μM cannabinoid. (**A**) Chemotaxis was measured as maximum cell index minus starting cell index and data are displayed as a fold change compared to cells migrating towards vehicle. Only CCL5, JWH133, HU308, L-759,656 and L-759,633 acted as chemoattractants. 2-AG was used at a concentration of 15 μM. Data are mean ± SEM, n = 3-26 biological replicates with 3-4 technical replicates per condition. (**B** and **C**) Representative traces of chemotaxis positive and chemotaxis negative CB_2_ agonists. Data show mean trace of 3-4 technical replicates per condition. Statistical analysis was conducted by one-way ANOVA with Dunnett’s multiple comparisons correction. ns P > 0.05, *** P < 0.001, comparing all samples to vehicle control. (**D**,**E**) Bio-gel elicited PECs (5 × 10^4^) were placed into a 96 well E-plate allowed to adhere for 2-3 hours at 37 °C, 5% CO_2_. Afterwards, cells were stimulated with either vehicle (0.3% DMSO) or cannabinoid. (**D**) Concentration response quantification demonstrates that L-759,656 caused cytoskeletal rearrangement in a concentration dependent manner, with an EC_50_ value of 7.3 μM. In contrast, (**D**) CP55,940, AM1241, (**E**) GP1a and WIN55,212-2 failed to elicit a response at any concentration (JWH133 curve is shown for comparison). Data are mean ± SEM, n = 3 biological replicates with 2-3 technical replicates per condition.

In order to examine the scope of CB_2_ agonist-induced chemotaxis, we expanded our list of ligands to include a range of widely used and commercially available compounds active at CB_2_. Based on our data with JWH133 and HU308, we selected 10 μM as the initial screening concentration, as this caused robust and reproducible chemotaxis. As shown in Fig. 3A, CB_2_ agonists fall into two distinct classes, with the majority (8/12 ligands tested) unable to induce chemotaxis. Nonetheless, alongside JWH133 and HU308, which significantly induced chemotaxis by 2 and 2.3 fold respectively (P < 0.001 versus vehicle), we discovered that the classical cannabinoids L-759,656 and L-759,633 also acted as macrophage chemoattractants (P < 0.001 versus vehicle). As shown in the representative traces, L-759,656 (10 and 1 μM) and L-759,633 (10 μM) gave robust increases in CI versus cells migrating towards vehicle alone (Fig. 3B,C). Kinetically, these compounds differed with respect to JWH133 and HU308 eliciting a slower chemotactic response, with peak CI reached between 2-2.5 hours. Furthermore, they were slightly less efficacious, inducing chemotaxis 1.7 (L-759,656) and 1.6 (L-759,633) fold versus vehicle. The aminoalkylindoles, (WIN55,212-2, JWH015, AM1241 and GW405833), the non-classical cannabinoid CP55,940, the tricyclic pyrazole GP1a, the endocannabinoid 2-AG and our novel CB_2_ agonist DIAS2 were all found to be negative for chemotaxis (Fig. 3A). The structures of all the compounds used in this study can be found in supplementary Fig. S1. Concentration response analysis conducted for AM1241, GP1a, GW405833 and CP55,940 (3-fold series from 30 μM to 100 nM) found that these agonists did not elicit chemotaxis at any concentration tested (data not shown). Real-time analysis of changes in cell morphology demonstrated that L-759,656 induced concentration dependent signalling (Fig. 3D), whereas the chemotaxis negative compounds CP55,950, AM1241 (Fig. 3D), GP1a and WIN55212-2 (Fig. 3E) did not induce signalling at any concentration tested. In summary, using both Boyden chamber and real-time chemotaxis assays we have shown that only a subset of CB_2_ agonists act as primary murine macrophage chemoattractants and only chemotaxis positive compounds were capable of inducing changes in cell morphology as measured by changes in electrical impedance.

### CB_2_ selective agonists induce primary chemotaxis independent of CB_2_

**Figure 4:**
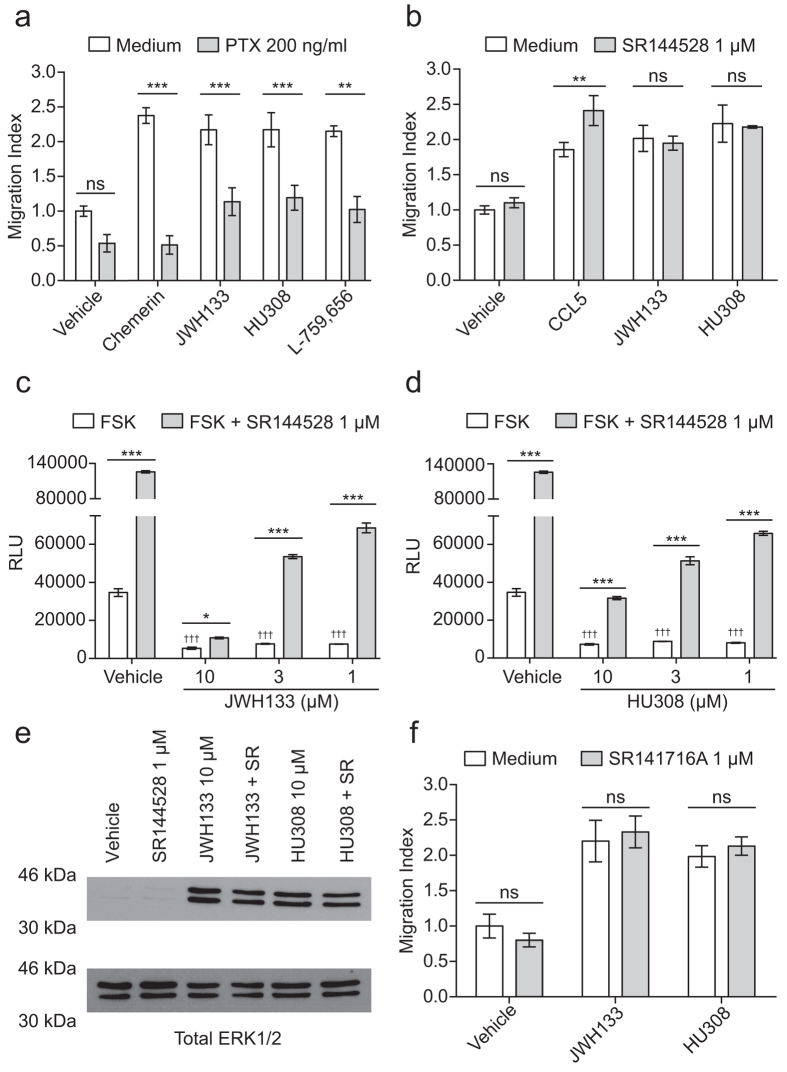
Chemotaxis towards CB_2_ selective agonists is blocked by pertussis toxin, but not by CB_2_ or CB_1_ inverse agonists. Bio-gel elicited PECs were incubated with either vehicle, (**A**) 200 ng/ml pertussis toxin (PTX) for 90 min or (**B**) 1 μM SR144528 for 30 min at 37 °C, 5% CO_2_. Following treatment, cells were allowed to migrate for 3-4 hours at 37 °C, 5% CO_2_ toward vehicle, 5 nM chemerin, 5 nM CCL5 or 10 μM cannabinoid. For experiments using SR144528, 1 μM was added in the lower chamber alongside the indicated chemoattractant. Data are mean ± SEM, (**A**) n = 4-11, (**B**) n = 3-10 biological replicates with 3-4 technical replicates per condition. (**C**,**D**) CHO-K1 cells expressing human CB_2_ were incubated for 30 min at 37 °C, 5% CO_2_ with either vehicle or SR144528 (1 μM - final assay concentration). Following treatment, vehicle, JWH133 (**C**) or HU308 (**D**) was added in assay buffer containing forskolin (FSK – 20 μM final assay concentration) to give the indicated final concentrations. The cells were then incubated for 30 min at 37 °C, 5% CO_2_ before the addition of the detection reagents. Data are mean ± SEM, n = 6. (**E**) Enriched macrophages were stimulated with either vehicle, or 10 μM cannabinoid in the presence and absence of 1 μM SR144528. Relative levels of phosphorylated ERK1/2 and total ERK1/2 were determined by western blotting. Blot is representative of n = 3 independent biological replicates. (**F**) Bio-gel elicited murine PECs were incubated with either vehicle or 1 μM SR141716A for 30 min at 37 °C, 5% CO_2_. Following treatment, cells were allowed to migrate for 3-4 hours at 37 °C, 5% CO_2_ toward vehicle or 10 μM cannabinoid. SR141716A at 1 μM was added in the lower chamber alongside the indicated cannabinoid. Data are mean ± SEM, n = 4 biological replicates with 3-4 technical replicates per condition. For all data, statistical analysis was conducted by two-way ANOVA with Sidak’s multiple comparisons correction. ns P > 0.05, * P < 0.05, ** P < 0.01, *** P < 0.001, for indicated comparisons. ††† P < 0.001, compared to vehicle + FSK alone.

To examine the reliance of CB_2_ agonist-induced migration on G protein activation, bio-gel elicited PECs were incubated with 200 ng/ml pertussis toxin (PTX) for 90 min prior to real-time chemotaxis analysis. Following pre-treatment, migration towards JWH133, HU308 and L-759,656 (all 10 μM) was significantly inhibited. Chemerin (5 nM), a chemoattractant protein^36^, was used as a PTX control. (Fig. 4A; Chemerin, JWH133 and HU308, P < 0.001; L-759,656, P = 0.0039), demonstrating that chemotaxis towards CB_2_ agonists absolutely requires G_i/o_ activation. We then used SR144528 (a widely used CB_2_ inverse agonist) to pharmacologically probe chemotaxis reliance on CB_2_ signalling. Surprisingly, pre-treatment with 1 μM SR144428 had no effect on macrophage chemotaxis towards JWH133 and HU308 (Fig. 4B). However, SR144528 did cause a significant 30% increase in PEC migration toward 5 nM CCL5 (P = 0.01). To rule out inactivity at CB_2_, we tested the ability of SR144528 to antagonise JWH133 and HU308-induced CB_2_ activation by measuring cAMP levels in CHO cells expressing the CB_2_. In our hands, JWH133 and HU308 acted as agonists at all concentrations (P < 0.001 versus vehicle plus Forskolin (FSK) alone), whereas SR144528 alone (1 μM) acted as an inverse agonist, significantly increasing FSK-induced cAMP levels (Fig. 4C,D; P < 0.001). Pre-incubation with SR144528 for 30 min prior to agonist addition significantly reversed JWH133 and HU308-induced CB_2_ activation at 10, 3 and 1 μM (Fig. 4C,D, respectively). To further study the ability of SR144528 to antagonise JWH133 and HU308-induced signalling we examined ERK1/2 phosphorylation in bio-gel elicited enriched macrophages, as CB_2_ agonism has been previously reported to activate the MAPK pathway^5^,^6^. Compared to vehicle, JWH133 and HU308 (10 μM) both caused substantial ERK1/2 phosphorylation, which SR144528 (1 μM) pre-treatment was unable to reverse (Fig. 4E). SR144528 alone gave no increase in the levels of phospho-ERK1/2. Total ERK1/2 levels confirmed equal protein loading across all samples. Although bio-gel elicited macrophages do not express CB_1_ mRNA (data not shown), we wanted to confirm that CB_1_ was not responsible for CB_2_ agonist-induced macrophage chemotaxis. Pre-treatment with 1 μM SR141716A (a CB_1_ receptor inverse agonist) for 30 min prior to real-time chemotaxis analysis had no effect on JWH133 or HU308-induced chemotaxis (Fig. 4F). SR141716A alone did not affect migration.

**Figure 5:**
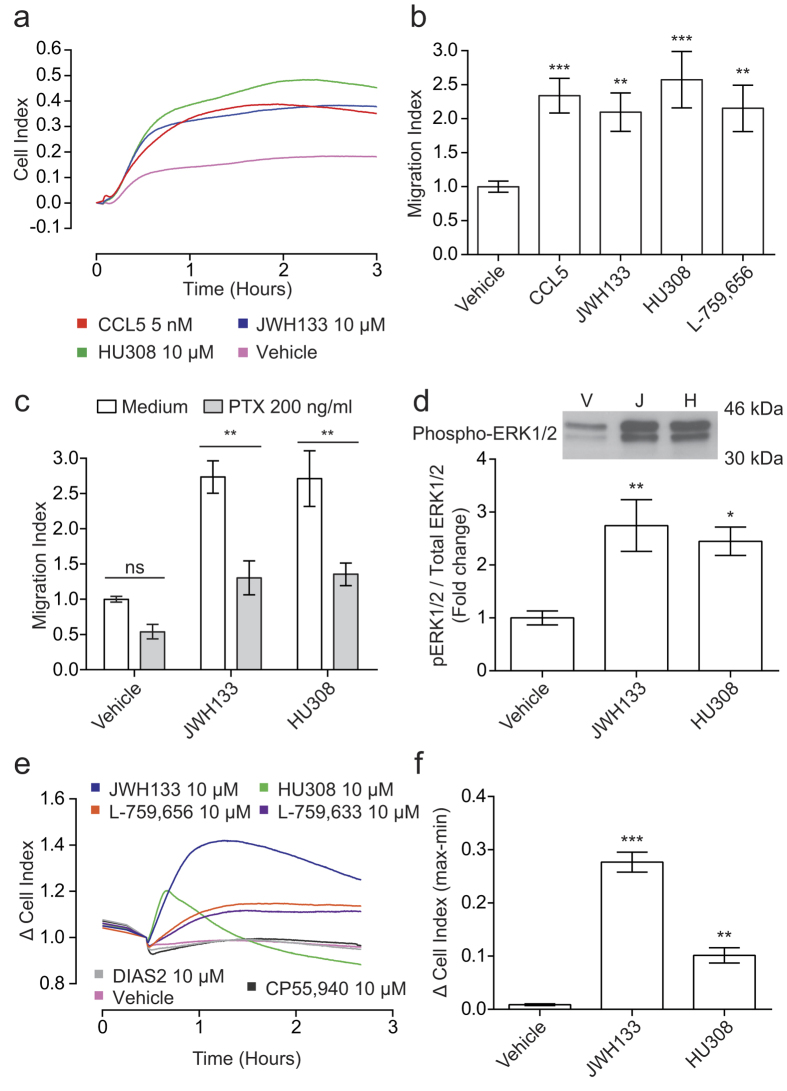
JWH133, HU308 and L-759,656 act as chemoattractants for CB_2_^-/-^ primary murine macrophages. Bio-gel elicited murine PECs from CB_2_^-/-^ mice were placed into the upper chamber of a CIM-16 plate and allowed to migrate for 3-4 hours at 37 °C, 5% CO_2_ toward vehicle (0.3% DMSO), 5 nM CCL5 or 10 μM cannabinoid. (**A**) Representative trace showing that the chemotaxis-positive CB_2_ agonists remain chemoattractants for CB_2_^-/-^ murine macrophages. (**B**) Quantification of chemotaxis demonstrates that CCL5 (5 nM), JWH133, HU308 and L-759,656 (all 10 μM) significantly induced CB_2_^-/-^ macrophage chemotaxis. Data are mean ± SEM, n = 5-15 biological replicates with 3-4 technical replicates per condition. (**C**) Pre-treatment of CB_2_^-/-^ bio-gel elicited macrophages with PTX (200 ng/ml, 90 min at 37 °C, 5% CO_2_) significantly reduced JWH133 and HU308-induced chemotaxis (both 10 μM). Data are mean ± SEM, n = 3 biological replicates with 2-3 technical replicates per condition. (**D**) Bio-gel elicited CB_2_^-/-^ macrophages were stimulated with either vehicle (0.3% DMSO) or 10 μM cannabinoid. Relative levels of phosphorylated ERK1/2 and total ERK1/2 were determined by western blotting and densitometric analysis confirmed that JWH133 and HU308 significantly elicited ERK1/2 phosphorylation. Data are mean ± SEM, n = 6 biological replicates. A representative phospho-ERK1/2 blot is shown as an insert. (**E**,**F**) Bio-gel elicited CB_2_^-/-^ macrophages were placed into a 96 well E-plate allowed to adhere for 2-3 hours at 37 °C, 5% CO_2_. Afterwards, cells were stimulated with either vehicle (0.3% DMSO) or cannabinoid (10 μM). (**E**) Representative trace showing the kinetics of CB_2_ agonist-induced cytoskeletal rearrangements. (**F**) Quantification demonstrates that JWH133 and HU308 significantly elicited cell spreading in CB_2_^-/-^ murine macrophages. Data are mean ± SEM, n = 4-5 biological replicates with 3-4 technical replicates per condition. For **B**,**D**,**F** statistical analysis was conducted by one-way ANOVA with Dunnett’s multiple comparisons correction. For **C** statistical analysis was conducted by two-way ANOVA with Sidak’s multiple comparisons correction * P < 0.05, ** P < 0.01, *** P < 0.001, comparing all samples to vehicle control.

To conclusively establish whether CB_2_ agonist-induced macrophage chemotaxis occurs independently of CB_2_, real-time chemotaxis analysis was conducted using bio-gel elicited PECs isolated from CB_2_ null mice (B6.129P2-Cnr2^tm1Dgen^/J mice from Jackson laboratories). Remarkably, 10 μM JWH133, HU308 and L-759,656 significantly induced CB_2_^-/-^ macrophage chemotaxis (Fig. 5B; P = 0.0045, P < 0.001 and P = 0.0027, respectively). As demonstrated by the real-time traces in Fig. 5A, JWH133 and HU308 retained similar kinetic chemotaxis profiles compared to WT cells. Comparable results were also obtained for L-759,656 and L-759,633 (data not shown). Pre-treatment of CB_2_^-/-^ macrophage with PTX significantly inhibited chemotaxis to both JWH133 and HU308 (Fig. 5C; P = 0.022 and P = 0.0034, respectively). Following this result, we wanted to examine whether JWH133 and HU308 could still activate intracellular signalling in the absence of CB_2_. Stimulation of bio-gel elicited enriched CB_2_^-/-^ macrophages with 10 μM JWH133 or HU308 resulted in a significant increase in the levels of phospho-ERK1/2 (Fig. 5D). Chemotaxis positive CB_2_ agonists retained their ability to induce cytoskeletal rearrangements in CB_2_^-/-^ macrophage, whereas chemotaxis negative agonists had no effect, replicating the WT results (Fig. 5E). Quantification of JWH133 and HU308 activity at 10 μM is shown in Fig. 5F (P < 0.001 and P = 0.0032, respectively compared to vehicle alone). Taken together, our results demonstrate that JWH133 and HU308 activate a G_i/o_-coupled receptor, which is not CB_2_, to elicit cytoskeletal rearrangement and directed macrophage migration.

### The candidate cannabinoid receptors GPR18 and GPR55 are not responsible for the off-target effects of JWH133 and HU308

**Figure 6:**
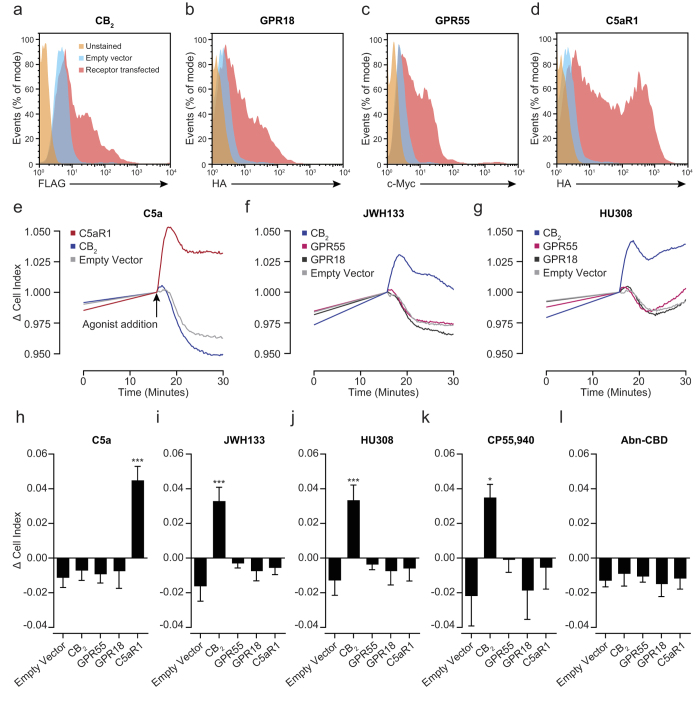
JWH133 and HU308 do not elicit cytoskeletal rearrangements in CHO cells expressing murine GPR18 or GPR55. CHO cells (1 × 10^6^) were mixed with 2 μg of empty or GPCR-containing vector, transfected via electroporation and left overnight at 37 °C, 5% CO_2_ to allow tagged GPCR expression. (**A-D**) GPCR surface levels were determined by flow cytometry and representative histograms are shown. Cells transfected with empty vector alone were used to determine non-specific antibody staining. (**A**) FLAG-CB_2_, (**B**) HA-GPR18 and c-Myc-GPR55 (**C**) all had similar expression levels. (**D**) HA-C5aR1 was used as a positive control and displayed high surface levels. (**E-L**) Transfected CHO cells were plated into a 96 well E-plate (25,000 cells/well) and left overnight at 37 °C, 5% CO_2_ to reach a stable baseline. Afterwards, cells were stimulated with either vehicle (0.3% DMSO), C5a (10 nM) or cannabinoid (10 μM). (**H**) Quantification of C5a-induced responses found that only C5aR1 transfected cells responded to C5a (10 nM; **E** – Representative trace). JWH133 (**F**,**I**), HU308 (**G**,**J**) and CP55,940 (**K**) only caused significant responses in CB_2_ transfected cells. Importantly, cells transfected with GPR18 or GPR55 did not respond to either JWH133 or HU308 (**I**,**J**). Abnormal cannabidiol (Abn-CBD; 10 μM) did not elicit a response in any of the GPCR transfected cells (**L**). Data are mean ± SEM, n = 3-6 biological replicates with 2-3 technical replicates per condition. Statistical analysis was conducted by one-way ANOVA with Dunnett’s multiple comparisons correction. * P < 0.05, *** P < 0.001, comparing all samples to empty vector transfected cells.

A body of literature exists implicating both GPR18 and GPR55 as potential cannabinoid receptors^37^,^38^,^39^,^40^. Additionally, activation of GPR55 by L-α-lysophosphatidylinositol causes the directed migration of human breast cancer cells^41^ and GPR18 has been shown to mediate 2-AG-induced chemotaxis of a microglial cell line^42^. Since both receptors are expressed in bio-gel elicited macrophages (Table 1), we set out to determine whether either was responsible for the off-target effects of JWH133 and HU308. N-terminal tagged receptor constructs were transfected into CHO cells using electroporation and surface receptor levels determined by flow cytometry after approximately 16 hours. In comparison to cells transfected with empty vector (blue histogram), CB_2_ (Fig. 6A), GPR18 (Fig. 6B) and GPR55 (Fig. 6C) transfected cells all had significant surface expression (red histogram), which was comparable across all three GPCRs (~50% positive). C5aR1 transfected CHO cells (selected as a positive control) had very high surface receptor levels (Fig. 6D; ~ 75% positive) and this was confirmed using an antibody that specifically recognises C5aR1 (data not shown). We then measured the ability of the chemotaxis positive agonists JWH133 and HU308, the chemotaxis negative ligand CP55,940 and C5a to induce cytoskeletal rearrangement in receptor transfected cells. Stimulation of transfected cells with 10 nM C5a (Representative ECIS trace – Fig. 6E) only elicited significant positive signalling in C5aR1 transfected cells (Fig. 6H; P < 0.0001, compared to empty vector transfected cells), confirming the assay worked as intended. Stimulation of cells with 10 μM JWH133 (Fig. 6F,I), HU308 (Fig. 6G,J) or CP55,940 (Fig. 6K) only caused significant cytoskeletal rearrangement in CB_2_ transfected cells (P < 0.0001 – JWH133; P = 0.0007 – HU308; P = 0.0186 – CP55,940; all in comparison to empty vector transfected cells). Abnormal cannabidiol (Abn-CBD) caused no response in any of the receptor transfected cells (Fig. 6L). Taken together, our data suggest that GPR18 and GPR55 are not responsible for the off-target effects of JWH133 and HU308.

**Figure 7:**
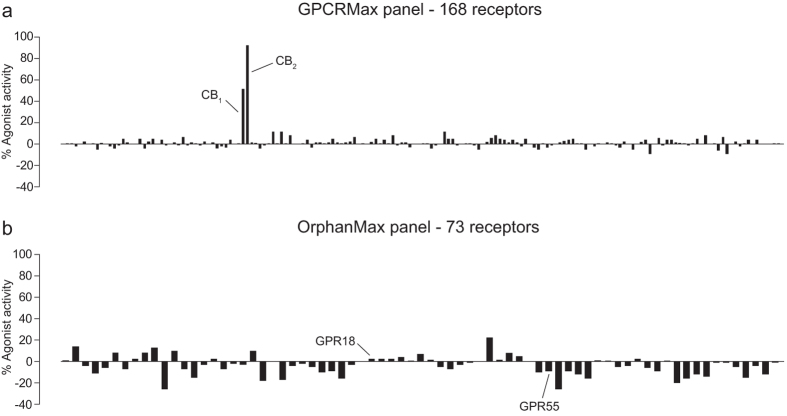
Out of 241 GPCRs, JWH133 only elicits β-arrestin recruitment at CB_1_ and CB_2_. JWH133 (10 μM) was tested for its ability to induce β-arrestin recruitment at 241 GPCRs, split over two panels, using the Discoverx PathHunter® eXpress β-Arrestin GPCR Assay system. (**A**) The GPCRMax panel was comprised of GPCRs with known and validated ligands. For this panel % Agonist activity is calculated as 100% x (mean of test sample - mean of vehicle control) / (mean MAX control ligand - Mean of vehicle control). JWH133 only caused β-arrestin recruitment at CB_1_ and CB_2_. (**B**) The OrphanMax panel was comprised of GPCRs without validated ligands. For this panel % Agonist activity is calculated as 100% x (mean of test sample - mean of vehicle control) / (mean of vehicle control). JWH133 did not elicit β-arrestin recruitment at either GPR18 or GPR55. Data are mean of technical duplicates.

In an attempt to find the receptor responsible for CB_2_ agonist-induced macrophage chemotaxis, we tested the ability of JWH133 (10 μM) to induce β-arrestin recruitment at 241 GPCRs using the Discoverx PathHunter® eXpress β-Arrestin GPCR Assay system. The screen was split into two panels, the first being GPCRMax, which contains CHO cells transfected with GPCRs that have well characterised agonists (Fig. 7A – 168 receptors) and the second being OrphanMax, which contains orphan GPCRs (Fig. 7B – 73 receptors). Both reassuringly and unfortunately, JWH133 only gave robust β-arrestin recruitment in CB_1_ and CB_2_ transfected CHO cells (Fig. 7A). Additionally, JWH133 was unable to induce β-arrestin recruitment in GPR18 and GPR55 transfected CHO cells (Fig. 7B). The full list of GPCRs tested and the assay results can be found as Supplementary data S1 online.

## Discussion

To our knowledge, this is the first study to address whether activation of CB_2_ causes the directed migration of primary murine macrophages. Using real-time measurements of both cell morphology and migration, we showed that only a subset of CB_2_ agonists act as chemoattractants for primary murine macrophages. Furthermore, we found that although CB_2_ agonist-induced chemotaxis was PTX sensitive, pharmacological inhibition or genetic ablation of CB_2_ had no effect on cellular signalling or macrophage migration. Therefore, this provides the first evidence that the widely used CB_2_ agonists JWH133, HU308, L-759,656 and L-759,633 have off-target effects at a non-CB_1_/CB_2_ G_i/o_-coupled GPCR; a finding that has wide ranging implications for the entire cannabinoid field. Finally, our data conclusively demonstrate that CB_2_ is not a macrophage chemoattractant receptor.

We are confident that throughout the study we were measuring macrophage chemotaxis, despite the fact that the bio-gel elicited PECs were comprised mostly of neutrophils. Without enrichment 4 × 10^5^ bio-gel elicited PECs were used per chemotaxis well and CI signals for JWH133 and HU308 peak at approximately 0.4. After macrophage enrichment of bio-gel elicited PECs, only 2 × 10^5^ cells are used per well, yet JWH133 and HU308 reach peak CIs of approximately 0.8 and retain similar kinetic profiles to normal bio-gel elicited PECs. Furthermore, thioglycollate elicited macrophages used at 2 × 10^5^ cells per well display chemotaxis kinetics and peak CI signals similar to the enriched macrophages. Importantly, the thioglycollate elicited macrophages did not contain neutrophils. Finally, neutrophils isolated from bone marrow only elicit a weak CI signal in real-time chemotaxis when migrating towards the potent neutrophil chemoattractant C5a. Therefore in bio-gel elicited PECs, neutrophils likely contribute only a negligible component to the observed CI signal as they do not score in the real-time chemotaxis system.

Initially we assumed that the ability of only a subset of CB_2_ agonists to induce macrophage chemotaxis was due to functional selectivity. This phenomenon, whereby ligands at the same receptor can activate distinct downstream signalling pathways^18^, is well documented for CB_2_ agonists acting at CB_2_^19^,^20^ and previous studies demonstrating that 2-AG acts as a chemoattractant, also found that that the synthetic CB_2_ agonists WIN55,212-2 and CP55,940 failed to elicit any migratory response^14^,^17^. However, this hypothesis was proved incorrect as CB_2_ agonist-induced macrophage chemotaxis was unaffected by pharmacological inhibition or genetic deletion of CB_2_. Instead our results demonstrate that the synthetic CB_2_ agonists JWH133, HU308, L-759,656 and L-759,633 activate at least one G_i/o_-coupled receptor distinct from CB_2_, to elicit directed cellular migration. This finding is not without precedent, as a non-CB_1_/CB_2_ receptor has been implicated in the negative regulation of fMLP-induced chemotaxis by endo and phytocannabinoids^27^. The profile of the receptor identified by McHugh *et al* does not match the putative GPCR from our study, however their observation demonstrates that novel receptors involved in chemotaxis modulation by cannabinoids may be more numerous than presently thought.

Chemical analysis of the agonists used in this study provides further support that JWH133, HU308, L-759,656 and L-759,633 have a specific mechanism at an off-target site. The chemotaxis positive compounds are all structurally related, being based on the phytocannabinoid Δ9-THC, and also have cLogP values in excess of 6.7 (Supplementary Fig. S1). As similar compounds normally have similar biological activity^43^, this implies that they share a common mode of action. However as CP55,940, which has a similar structure and lipophilicity to the chemotaxis positive compounds, was unable to induce directed migration, it is unlikely that the ability of only some CB_2_ ligands to induce chemotaxis relies solely on their physicochemical properties. Instead we conclude that CP55,940 likely violates the steric constraints of the off-target site rendering it inactive and unable to induce directed migration.

Currently the identity of the receptor responsible for CB_2_ agonist-induced chemotaxis remains unknown, however we initially ruled out CB_1_ as it was not expressed on bio-gel elicited macrophages and SR141617A had no effect on CB_2_ agonist-induced chemotaxis. We next focussed on GPR55 and GPR18 as potential candidates, as both bind numerous cannabinoid compounds^37^,^39^,^40^ and have been shown to mediate directed cellular migration^38^,^41^,^42^. We elected to use electrical impedance measurement of cell morphology changes as this system correlated strongly with chemotaxis and also allows for multiple receptors and agonists to be tested simultaneously. Nonetheless, JWH133 and HU308 were unable to induce cytoskeletal rearrangements in CHO cells expressing GPR18 or GPR55. Furthermore, JWH133 did not elicit β-arrestin recruitment at either receptor. Consequently, both were ruled out as the GPCR in question.

It is important to note that the negative result from the β-arrestin screen does not conclusively prove that none of the 241 GPCRs tested are responsible for the off-target effects of the synthetic CB_2_ agonists and should be viewed with respect to the following caveats. Firstly, functional selectivity may mean that JWH133 does not cause β-arrestin recruitment at this particular GPCR. Secondly, as human GPCRs were used for the screen (as murine versions were not available) species differences between CB_2_ pharmacology, which have been well documented *in vitro*^44^,^45^,^46^, could explain the negative result. However, as JWH133 has already been shown to act as a chemoattractant for human monocytes, at the same concentrations used in this study, we assumed that this would likely not be an issue^13^. Finally, recent work has shown that established CB_2_ ligands can behave differently in primary tissue as opposed to recombinant cellular systems as in human, rat and mouse spleen, AM1241 behaves as an agonist, whereas in CHO cells overexpressing human CB_2_ it acts as an inverse agonist^47^. Therefore although the CB_2_ ligands are acting as agonists for the novel GPCR in primary murine macrophages, they may display different pharmacology in the *in vitro* system used for the β-arrestin screen.

Regardless of the exact nature of the receptor, we believe our finding that JWH133, HU308, L-759,656 and L-759,633 are more promiscuous than currently thought has wide-ranging implications for the cannabinoid field as a whole. Numerous endogenous and plant derived cannabinoids have been shown to activate a multitude of receptors other than CB_1_ or CB_2_, however to our knowledge this is the first report that these synthetic agonists have off-target effects of functional consequence in disease relevant primary cells. This is of importance as JWH133 and HU308 are among the agonists most frequently used to assess the selective activation of CB_2_^2^. However their reputation as selective agonists, cemented by historical compound characterisation for CB_1_/CB_2_ activity in transfected cell systems overexpressing either cannabinoid receptor, has meant that many studies do not employ stringent controls when using these ligands. A cursory search of the literature found multiple examples of JWH133 and HU308 being used in both primary cell assays and *in vivo* disease models without receptor antagonism or CB_2_ deletion to confirm CB_2_ dependence^48^,^49^,^50^,^51^,^52^,^53^,^54^,^55^,^56^,^57^. The off-target effects of JWH133 and HU308 may mean that results obtained in these studies have been erroneously attributed solely to the activation of CB_2_. Additionally, many of the above models have a strong inflammatory component and as macrophages are key players during inflammation^58^,^59^, the macrophage localised GPCR implicated in this study has likely confounded observed results. Indeed this receptor may even explain current findings in the literature. For example, the previously mentioned ability of JWH133 to be a chemoattractant for human monocytes was not confirmed to absolutely depend on CB_2_^13^. In light of our data we speculate that, rather than CB_2_, the novel receptor implicated in our study may be responsible for JWH133-mediated chemotaxis of human monocytes.

In summary our work emphasises the importance of using primary cells wherever possible, as although *in vitro* cell systems have undoubtedly proved crucial for our understanding of CB_1_ and CB_2_ pharmacology, by their very nature they will not elucidate the targets and pharmacology of CB_2_ agonists in physiologically relevant settings. Furthermore, it is critical that genetic knockout alongside pharmacological blockade should be used wherever possible when seeking evidence of a CB_2_ specific effect using primary cells, tissues or *in vivo* disease models.

## Additional Information

**How to cite this article**: Taylor, L. *et al.* Primary Macrophage Chemotaxis Induced by Cannabinoid Receptor 2 Agonists Occurs Independently of the CB_2_ Receptor. *Sci. Rep.* **5**, 10682; doi: 10.1038/srep10682 (2015).

## Supplementary Material

Supplementary Figure S1

Supplementary Data S1

## Figures and Tables

**Table 1:** Bio-gel elicited macrophages express CB_2_, GPR55 and GPR18.

**Gene**	**Mean Ct value**	**SEM**
**Actin**	14.09	0.305
**CB**_**2**_	22.52	0.5536
**GPR55**	25.72	0.1437
**GPR18**	22.01	0.3132

Expression of CB_2_, GPR55 and GPR18, alongside the reference gene actin, was determined by real-time PCR using 500 ng of cDNA per reaction with the following thermal profile: 95 °C for 5 min, 40 cycles of 95 °C for 30 s, 60 °C for 20 s, 72 °C for 30 s and a final step of 72 °C for 5 min. Mean threshold cycle (Ct) value and SEM were calculated from n = 4 biological replicates.
