# Supplementary material for: Primary Macrophage Chemotaxis Induced by Cannabinoid Receptor 2 Agonists Occurs Independently of the CB2 Receptor
Source: Sci Rep. 2015 Jun 2;5:10682. doi: 10.1038/srep10682 (PMC4451551; doi:10.1038/srep10682)
Supplement: Supplementary Figure S1 [file srep10682-s1.pdf]

Supplemental data for:

Primary macrophage chemotaxis induced by cannabinoid receptor 2 agonists occurs independently of the CB_2 receptor

Lewis Taylor, Ivy Christou, Theodore S. Kapellos, Alice Buchan, Maximillian H. Brodermann, Matteo Gianella-Borradori, Angela Russell, Asif J. Iqbal and David R. Greaves

Scientific Reports

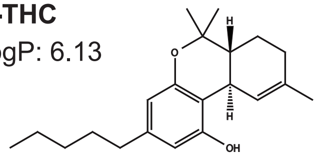

Δ9-THC
cLogP: 6.13

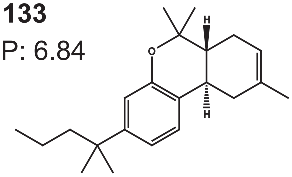

JWH133
cLogP: 6.84

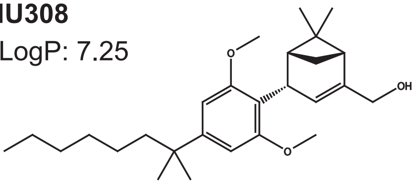

HU308
cLogP: 7.25

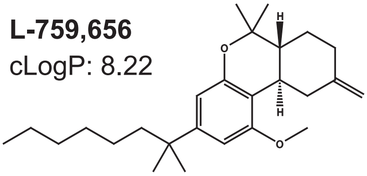

L-759,656
cLogP: 8.22

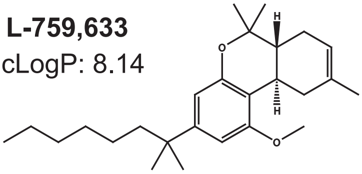

L-759,633
cLogP: 8.14

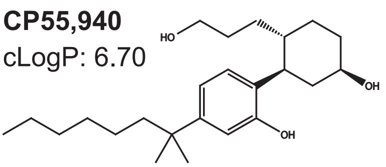

CP55,940
cLogP: 6.70

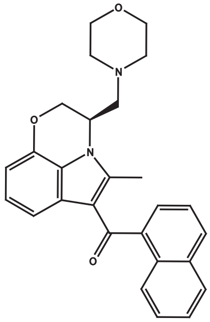

WIN55,212-2
cLogP: 4.43

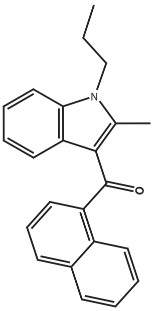

JWH015
cLogP: 5.26

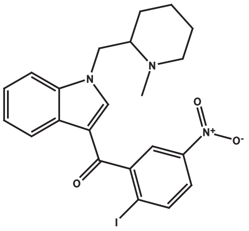

AM1241
cLogP: 3.08

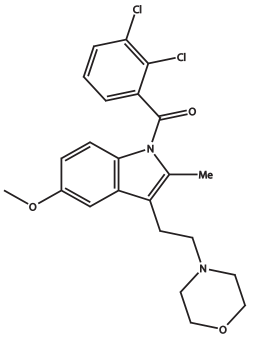

GW405833
cLogP: 5.12

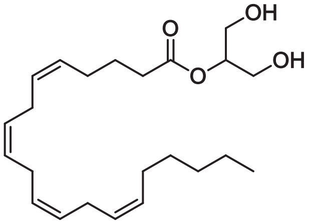

2-AG
cLogP: 6.21

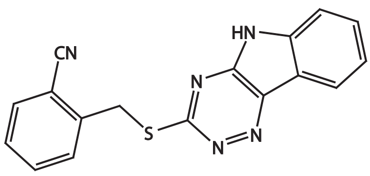

DIAS2
cLogP: 3.07

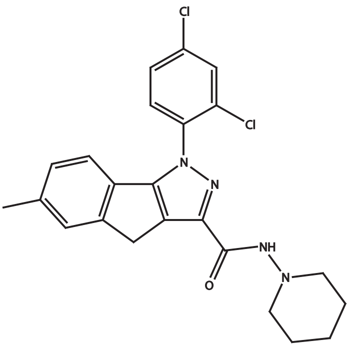

GP1a
cLogP: 3.96

Supplementary Figure S1
